# Long‐term survival of a patient with refractory advanced adrenocortical carcinoma after combination chemotherapy with paclitaxel and carboplatin plus mitotane

**DOI:** 10.1002/iju5.12458

**Published:** 2022-04-30

**Authors:** Yuki Kobayakawa, Shuzo Hamamoto, Hideyuki Kamisawa, Shinsuke Okada, Kazumi Taguchi, Taku Naiki, Atsushi Okada, Keiichi Tozawa, Takahiro Yasui

**Affiliations:** ^1^ Department of Nephro‐Urology Nagoya City University Graduate School of Medical Sciences Nagoya Japan; ^2^ Department of Urology Kainan Hospital Yatomi Japan; ^3^ Department of Urology Gyotoku General Hospital Chiba Japan

**Keywords:** adrenocortical carcinoma, carboplatin, liver neoplasms, paclitaxel

## Abstract

**Introduction:**

The prognosis of patients with unresectable adrenocortical carcinoma is poor. Mitotane is the first‐line treatment for this disease, and etoposide/doxorubicin/cisplatin/mitotane therapy is recommended as first‐line chemotherapy in unresponsive cases. We present a case of long‐term survival following combination chemotherapy with paclitaxel and carboplatin plus mitotane to manage mitotane‐refractory advanced adrenocortical carcinoma.

**Case presentation:**

A 49‐year‐old woman with a left adrenal tumor, lymph node metastasis around the aorta, and multiple liver metastases was treated with mitotane. The disease progressed despite mitotane therapy; thus, combination chemotherapy with paclitaxel and carboplatin plus mitotane was administered for 9 months. Primary adrenal resection was performed after the liver metastasis had completely dissapeared. She has remained alive for 20 years since her initial diagnosis while undergoing mitotane therapy.

**Conclusion:**

In this case, combination chemotherapy with paclitaxel and carboplatin plus mitotane effectively controlled advanced adrenocortical carcinoma.

**Abbreviations & Acronyms**
ACC: adrenocortical carcinoma
CT: computed tomography
EDP‐M: etoposide/doxorubicin/cisplatin/mitotane therapy
TJ‐M: paclitaxel and carboplatin plus mitotane

**Keynote message**

TJ‐M combination chemotherapy was administered to a patient with a left adrenal tumor, lymph node metastasis around the aorta, and multiple liver metastases. After remission of the liver metastases following nine courses of combination chemotherapy, the primary adrenal lesion was surgically resected along with the left kidney. At the last follow‐up, it was revealed that she has remained alive for 20 years since her initial diagnosis.

## Introduction

ACC is a rare tumor with an incidence rate of 0.7–2.0 cases/million people/year.^1^ Patients with locally advanced or metastatic ACC, those unresponsive to surgery, and those with limited treatment options tend to have poor prognoses. According to the expert consensus guidelines on the management of ACC, the 5‐year overall survival rate of those with distant metastases is <15%.^2^ In general, first‐line therapy in patients with advanced/metastatic disease is mitotane alone or mitotane plus combination chemotherapy of etoposide, doxorubicin, and cisplatin.^2^, ^3^ However, no guidelines regarding chemotherapy for mitotane‐refractory advanced ACC had been defined at the time of this case (20 years ago from now). We present a case that has been overlooked for 20 years in which combination chemotherapy with TJ‐M was effective against mitotane‐refractory advanced ACC.

## Case report

**Fig. 1:**
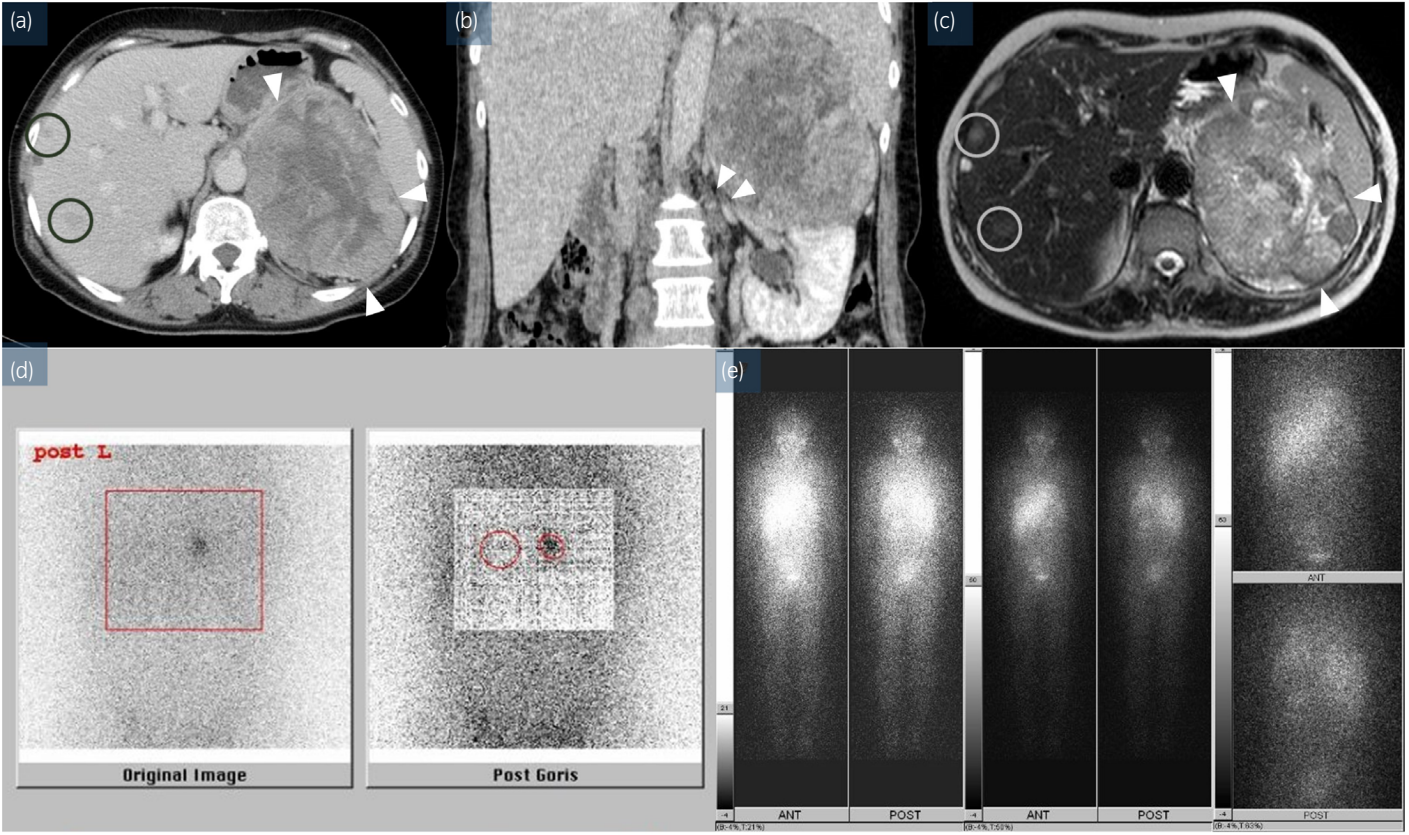
Imaging findings. Enhanced CT showing (a) a retroperitoneal tumor (arrowhead, 95 × 123 × 132 mm) and metastatic liver tumors (circle), and (b) lymphadenopathy of the hilar region of the kidney (arrowhead). (c) Magnetic resonance imaging (T2WI) showing a retroperitoneal tumor (arrowhead) and metastatic liver tumors (circle). (d) ^131^I‐adsterol scintigraphy showing no signal accumulation in the left adrenal gland (circle). (e) ^131^I‐MIBG scintigraphy showing no signal accumulation in the retroperitoneal tumor. [Colour figure can be viewed at wileyonlinelibrary.com]

**Fig. 2:**
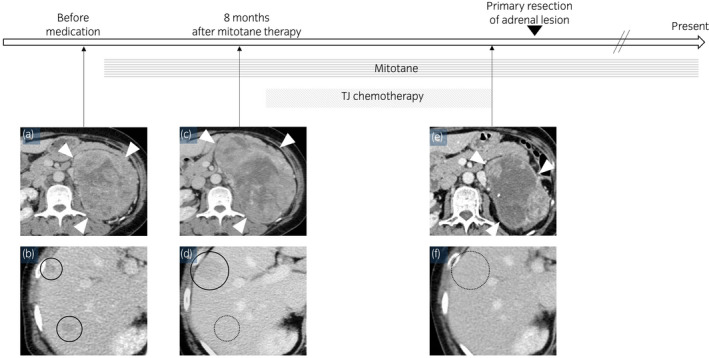
Therapeutic schedule and imaging follow‐up. (a, b) Enhanced CT performed at the initial medical examination showing a retroperitoneal tumor measuring 95 × 123 × 132 mm (arrowhead) and metastatic liver tumor (circle). (c, d) Tumor progression in the adrenal and liver lesions (arrowhead and circle) 8 months after mitotane therapy initiation. (e, f) Complete remission was achieved for the liver metastasis (circle). The adrenal tumor shrank, and internal necrosis spread after 9 cycles of the chemotherapy (arrowhead). [Colour figure can be viewed at wileyonlinelibrary.com]

**Fig. 3:**
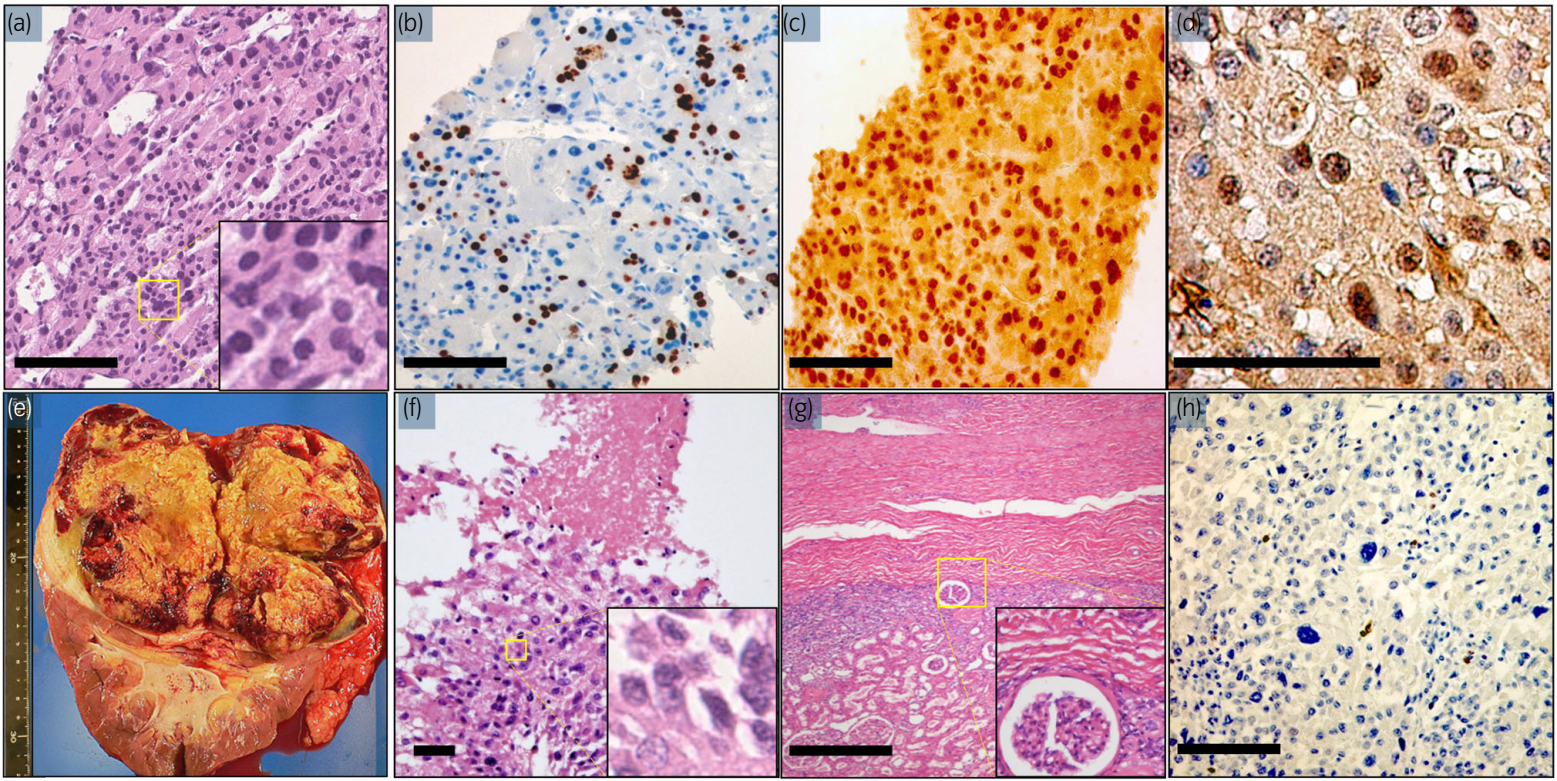
Pathological findings. (a–d) Biopsy sample of the adrenal tumor. (a) Tumor cells with monotonous morphology and large, centrally located nuclei and abundant cytoplasm. Focal tumor necrosis was present (hematoxylin and eosin staining). (b) The Ki‐67 labeling index is 30% at the hot spot. (c) Positive expression of steroidogenic factor 1 (hematoxylin and eosin staining, ×100). (d) Positive expression of caveloin‐1 (e). A piece of tissue weighing 968 g. (f) Viable tumor cells with pleomorphic nuclei and eosinophilic cytoplasm with (g) no tumor infiltration of the kidney. (h) The Ki‐67 labeling index decrease to 4% after the TJ‐M therapy. [Colour figure can be viewed at wileyonlinelibrary.com]

The patient was a 49‐year‐old woman with uterine cancer. CT revealed a left adrenal tumor, enlarged aortic lymph nodes, and multiple liver tumors (Figs 1a–c and 2e,f). Serum hormone and tumor marker levels were normal, and no signal accumulation was observed in the left adrenal glands on ^131^I‐adsterol scintigraphy (Fig. 1d) and a retroperitoneal tumor on ^123^I‐meta‐iodobenzylguanidineI scintigraphy (Fig. 1e). Ultrasonography‐guided percutaneous biopsy of the left adrenal mass was performed, which revealed that the tumor cells contained abundant granular eosinophilic cytoplasm, and the Weiss score^4^ was 4 points (out of 9), exhibiting high mitotic rate, atypical mitoses, eosinophilic cytoplasm, and coagulation necrosis (Fig. 3a). The Ki‐67 labeling index was 30% at the hot spot (Fig. 3b). Tumor cells were immunohistochemically positive for steroidogenic factor 1 (Fig. 3c) and caveolin‐1 (Fig. 3d).

The patient was diagnosed with primary nonfunctional ACC, T2N1M1, and ENSAT stage IV based on the diagnostic imaging and histopathological findings. As the adrenal gland tumor was too large for curative resection, the patient was treated with mitotane (1500 mg/day) orally as first‐line therapy, but the dose was reduced to 500 mg/day due to drug‐induced liver dysfunction. Eight months after mitotane administration, CT revealed progression of the adrenal mass and liver metastasis (Fig. 2c,d).

She then received systemic chemotherapy consisting of TJ‐M. A total of 70 mg/m^2^ paclitaxel was administered weekly via intravenous infusion on days 1, 8, and 15, while carboplatin (area under the curve, 5) was administered every 28 days starting from day 1. The patient received premedication for paclitaxel‐associated hypersensitivity reactions, consisting of dexamethasone (8 mg), diphenhydramine (50 mg), and ranitidine (50 mg). After three cycles, she achieved complete remission of the liver metastasis, and the adrenal tumors reduced in size. Nine courses of combination chemotherapy were administered following this regimen without any progression (Fig. 2e,f). However, chemotherapy could not be continued due to severe bone marrow suppression. Thus, the primary adrenal lesion was surgically resected. The adrenal glands along the left kidney were also removed (Fig. 3e). Histological findings revealed viable tumor cells with pleomorphic nuclei and eosinophilic cytoplasm with no tumor infiltration of the kidney (Fig. 3f,g). The Ki‐67 labeling index decreased to 4% after TJ‐M therapy (Fig. 3h). At the last follow‐up, she had been alive for 20 years since her initial diagnosis and treatment with oral mitotane (500 mg/day).

## Discussion

Mitotane is the only drug approved in Japan for patients with locally advanced, unresectable, metastatic ACC. The first‐line therapy in patients with metastatic ACC is mitotane alone or in combination with chemotherapy. Expert consensus guidelines recommend a combination of EDP‐M as first‐line treatment.^3^ A phase III randomized trial reported in 2012 revealed that the progression‐free survival of patients treated with EDP‐M was superior to that of patients treated with streptozocin plus mitotane (just failing to attain statistical significance).^5^ Gemcitabine plus capecitabine or streptozotocin may be considered as other appropriate therapies.^6^, ^7^

At the time of this case, no guidelines regarding chemotherapy for mitotane‐refractory advanced ACC had yet been defined. Although there was no effective clinical experience or prior literature at the time, TJ‐M chemotherapy was selected for patient. Taxanes have previously been suggested as viable treatment options for ACC. Fallo *et al*. reported the antiproliferative effect of paclitaxel on a human steroid‐secreting NCI‐H295 adenocarcinoma cell line in vitro. Paclitaxel caused dose‐dependent inhibition of cell proliferation and apoptosis, as it led to neoplastic cell death.^8^ The combination of carboplatin and paclitaxel has been commonly used in patients with breast, ovarian, and non‐small cell lung cancers, but for ACC, only one case after ours has been reported in the literature.^9^ Paclitaxel, carboplatin, and etoposide with mitotane was used in one case.^10^ However, both of them did not show effectiveness. Analysis of immunohistochemical markers expressed in sarcomatoid ACCs revealed that epithelial‐mesenchymal transition‐related markers, including E‐/P‐/N‐cadherin, MMP‐2/−9, and caveolin‐1, may play a role in the poor prognosis.^11^ In the present case as well, the tumor cells were positive for caveolin‐1. As the expression of caveolin‐1 reportedly enhances the paclitaxel‐mediated cytotoxicity of breast cancers,^12^ TJ‐M chemotherapy was hypothesized to be effective for this ACC case.

This patient has been alive for 20 years since her initial diagnosis due to continuous oral mitotane administration. Few cases of long‐term survival following advanced ACC have been reported to date. Based on the ENSAT staging, 5‐year survival rates are 82%, 61%, 50%, and 13% for patients with Stage I, II, III, and IV disease, respectively.^13^ To achieve long‐term survival, complete excision of the primary lesion with adjacent organ resection and adjuvant treatment with oral mitotane is recommended due to the high incidence of recurrence of this tumor.^14^ The blood concentration of mitotane must be adjusted to 14–20 mg/L to balance the efficacy and toxicity.^15^ In this case, the patient received a very low dose of 500 mg/day mitotane as adjuvant therapy, which was much less than the recommended dose in previous reports. We certainly do not know whether low‐dose mitotane therapy was effective in this case. However, her long‐term survival suggests that both complete surgical resection and continued low‐dose mitotane contribute to the suppression of tumor recurrence. As the ESMO‐EURACAN Clinical Practice Guideline in 2020 did not recommend it beyond 5 years due to the low number of ACC patients who recurred after 5 years, low dose mitotane may be stopped in the future.^2^

## Conclusion

To our knowledge, this is the first case of a patient with mitotane‐refractory advanced ACC achieving long‐term survival with combined chemotherapy comprising of TJ‐M.

## Author contributions

Yuki Kobayakawa: Conceptualization; data curation; funding acquisition; methodology; resources; writing – original draft; writing – review and editing. Shuzo Hamamoto: Conceptualization; data curation; funding acquisition; methodology; project administration; supervision; visualization; writing – original draft; writing – review and editing. Hideyuki Kamisawa: Conceptualization; data curation; funding acquisition; methodology; resources; supervision; writing – review and editing. Shinsuke Okada: Conceptualization; investigation; methodology. Kazumi Taguchi: Conceptualization; funding acquisition; methodology; supervision; writing – review and editing. Taku Naiki: Conceptualization; data curation; funding acquisition; resources; supervision; writing – review and editing. Atsushi Okada: Conceptualization; data curation; methodology; resources; supervision; writing – review and editing. Keiichi Tozawa: Conceptualization; funding acquisition; writing – review and editing. Takahiro Yasui: Conceptualization; funding acquisition; methodology; supervision; writing – review and editing.

## Conflict of interest

The authors declare no conflict of interest.

## Approval of the research protocol by an Institutional Reviewer Board

The protocol for this research project was approved by a suitably constituted Ethics Committee of Nagoya City University Graduate School of Medical Sciences, IRB No. 60‐19‐0088. and it conforms to the provisions of the Declaration of Helsinki.

## Informed consent

Informed consent was obtained from the patient.

## Registry and the Registration No. of the study/trial

N/A.
